# *Paracaesicola nanshaensis* n. gen., n. sp. (Monogenea, Microcotylidae) a gill parasite of *Paracaesio sordida* (Teleostei, Lutjanidae) from the South China Sea

**DOI:** 10.1051/parasite/2020031

**Published:** 2020-05-15

**Authors:** Zi-Hua Zhou, You-Zhi Li, Lin Liu, Xue-Juan Ding, Kai Yuan

**Affiliations:** Guangdong Provincial Key Laboratory for Healthy and Safe Aquaculture, College of Life Science, South China Normal University 510631 Guangzhou PR China

**Keywords:** Monogenea, Microcotylidae, *Paracaesicola nanshaensis* n. gen. n. sp., *Paracaesio sordida*, The South China Sea

## Abstract

*Paracaesicola* n. gen., is erected herein to accommodate a new microcotylid species, *Paracaesicola nanshaensis* n. sp., collected from the Yongshu Reef, South China Sea. This species is the first monogenean to be recorded from the gills of *Paracaesio sordida*. The new species is characterized by the following features: (i) haptor short, with clamps arranged in two equal bilateral rows; (ii) testes numerous, arranged in two roughly alternating longitudinal rows, extending into the haptor; (iii) genital atrium armed with 16 robust spines, which are vertically arranged on top of the sausage shaped muscular male copulatory organ; and (iv) single vagina, bottle-shaped, with a distinctly bulbous vaginal atrium. The terminals of the reproductive system discriminate *Paracaesicola* n. gen. from all other genera in the Microcotylidae. Molecular phylogenetic analyses, based on partial 28S rDNA, places *Paracaesicola nanshaensis* n. sp. within the microcotylid clade, but its sequence differs from all known available microcotylid sequences.

## Abstract (fr)

*Paracaesicola* n. gen. est érigé ici pour accueillir une nouvelle espèce de Microcotylidae, *Paracaesicola nanshaensis* n. sp., collectée sur le récif de Yongshu, mer de Chine méridionale. Cette espèce est le premier monogène signalé des branchies de *Paracaesio sordida*. La nouvelle espèce est caractérisée par : (i) hapteur court, avec des pinces disposées en deux rangées bilatérales égales ; (ii) testicules nombreux, disposés en deux rangées longitudinales à peu près alternées, s’étendant jusqu’au hapteur ; (iii) atrium génital armé de 16 épines robustes, disposées verticalement au-dessus de l’organe copulateur mâle, musculaire et en forme de saucisse ; et (iv) vagin unique, en forme de bouteille, avec un atrium vaginal nettement bulbeux. Les parties terminales du système reproducteur distinguent *Paracaesicola* n. gen. de tous les autres genres de Microcotylidae. Les analyses phylogénétiques moléculaires, basées sur l’ADNr 28S partiel, placent *Paracaesicola nanshaensis* n. sp. au sein du clade des Microcotylidae, mais sa séquence diffère de toutes les séquences de Microcotylidae disponibles.

## Introduction

Microcotylidae Taschenberg, 1879 is the most speciose family in the Polyopisthocotylea monogeneans, and most species in this family parasitize marine teleost fishes. Tripathi [33] and Dillon & Hargis [10] proposed early arrangements of the species in this family. Unnithan [34] proposed a taxonomic rearrangement of Microcotylidae, dividing the family into four subfamilies and 18 genera. However, Mamaev [28] considered that Unnithan’s work could result in additional confusion, and rejected all Unnithan’s subfamilies, recognising only five of his 13 new genera. This rearrangement was revised again by Mamaev [29], who proposed eight subfamilies and 39 genera. Several genera were subsequently added to the family, such as *Neobivaginopsis* Villalba, 1987, *Tinrovia* Mamaev, 1987, *Serranicotyle* Maillard, Euzet & Silan, 1988, *Sciaenacotyle* Mamaev, 1989, *Synocoelicotyloides* Mamaev & Brashovian, 1989, *Paranaella* Kohn, Baptista-Farias & Cohen, 2000 and *Omanicotyle* Yoon, Al-Jufaili, Freeman, Bron, Paladini & Shinn, 2013 [14, 20, 27, 30, 39]. Several new species of Microcotylidae have recently been described [2, 4–6, 8, 13, 21]. At present, the Microcotylidae includes more than 200 species in 53 genera [37], 29 species in 13 genera are known from China [40, 42].

Coral reefs are well known for their remarkable biodiversity. Many monogenean species have been described from the coral reefs of the Hawaiian Islands, the Great Barrier Reef, and New Caledonia [17, 18, 35, 38]. The Nansha Islands of the South China Sea are a typical coral reef ecosystem, from which numerous coral reel fishes have been recorded [25]. However, the diversity of monogeneans in this region is poorly known. During an ongoing investigation of the monogenean fauna from the South China Sea, specimens of an undescribed species of Microcotylidae were collected from the Yongshu Reef. The morphological features of this new species are described herein, and a new genus is proposed to accommodate this new species*.*

## Materials and methods

### Specimen collection and morphological analysis

**Figure 1:**
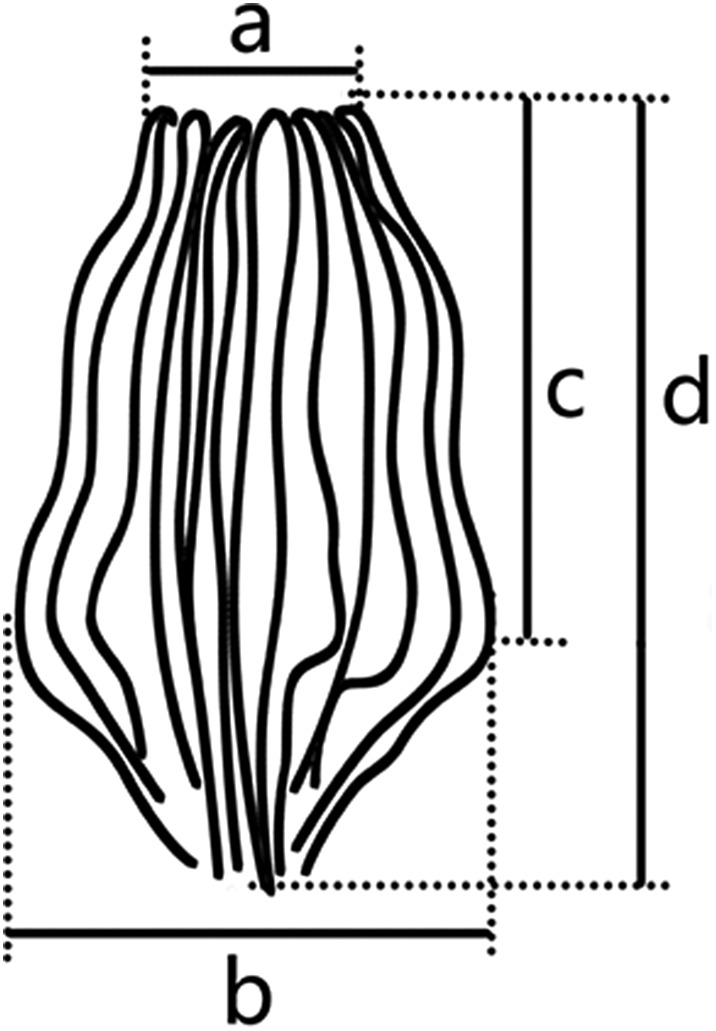
Method of measurements of genital atrium spines.

Fishes were caught by line angling off the Yongshu Reef in the South China Sea from March to May, 2016. Parasites were collected and treated as described by Zhang et al. [41]. Twelve parasitic specimens were preserved in 70% ethanol for subsequent staining with acetic carmine, four were mounted in Berlese fluid for the morphological study of the hard parts, and two were digested with proteinase K (20 μg/μL) for 30 min at room temperature for the analysis of genital spines. The remaining specimens were fixed in 95% ethanol for DNA extraction. Illustrations were drawn with the help of an Olympus LB (Olympus Corporation, Japan), scanned, and processed with Photoshop CS4.0 (Adobe, USA). Specimens were measured using Olympus DP22 software. All measurement ranges are given in micrometres, followed by the mean and the number of specimens studied (*n*) in parentheses. The method for measurement of genital atrium spines is shown in Figure 1.

### Sequences of monogeneans

Total genomic DNA was extracted using the TIANamp Marine Animals DNA Kit (Tiangen, China), following the manufacturer’s instructions. We amplified the C1–D2 fragment of the 28S ribosome RNA subunit with PCR using previously published primer pairs (C1F: 5′ – ACCCGCTGAATTTAAGCAT – 3′ and D2R: 5′ – TGGTCCGTGTTTCAAGAC – 3′) [11]. Each 25 μL PCR contained 12.5 μL Master Mix (2x), 7.5 μL ddH_2_O, 1 μL of each primer, and 3 μL genomic DNA. The PCR cycling conditions were initial denaturation at 94 °C for 5 min; followed by 35 cycles of 94 °C for 1 min, 56 °C for 45 s, and 72 °C for 1 min; and a final elongation at 72 °C for 10 min. PCR products were confirmed by 1% agarose gel electrophoresis, and sequenced by the Sangon Biotech Company (Guangzhou, China). The sequences obtained were analyzed using DNAMAN 7.0 and Sequencher 5.0 (Gene Codes Corp.) software, compared to the GenBank database content with BLAST, and deposited in GenBank under accession number MH700264, with a final length of 881 bp.

### Trees of monogeneans and distances

A tree was constructed from our new sequence of *Paracaesicola nanshaensis* n. sp. and 28S sequences of microcotylids available in GenBank; a sequence of *Polystomoides asiaticus* Rohde, 1965 was used as the outgroup. The dataset included 29 nucleotide sequences (Table 1). There were 855 positions in the dataset (including gaps). After estimating the best model with MEGA7 [22], the tree was inferred using the maximum likelihood (ML) method based on the general time reversible model with gamma distribution (GTR + G) in MEGA7 [31], with 1000 replications. The neighbour-joining (NJ) method was also used for comparison in MEGA7. Distances between sequences (Kimura-2 parameter distances) were computed from the same dataset with MEGA7 [19].

**Table 1:** Species of the monogeneans used in the molecular analysis.

Parasite	Host	Location	Accession no.	Reference
*Paracaesicola nanshaensis* n. sp.	*Paracaesio sordidus* (Abe et Shinohara)	China	MH700264	Present study
*Diplostamenides sciaenae* (Goto, 1894) Mamaev, 1986	–	China	FJ432589	Direct submission
*Diplostamenides* sp.	*Johnius belangerii* (Cuvier)	China	MH700263	Direct submission
*Cynoscionicola branquialis*	*Umbrina xanti* Gill	Mexico	AF382050	[32]
*Microcotyle erythrini* Van Beneden & Hesse, 1863	*Pagellus erythrinus* (Linnaeus)	France	AM157221	[3]
*Microcotyle arripis* Sandars, 1945	*Arripis georgianus* (Valenciennes)	Australia	GU263830	[7]
*Microcotyle sebastis* Goto, 1894	*Sebastes* sp.	UK	AF382051	[32]
*Omanicotyle heterospina* (Mamaev & Parukhin, 1974) Yoon, Al–Jufaili, Freeman, Bron, Paladini & Shinn, 2013	*Argyrops spinifer* (Forsskål)	Oman	JN602095	[39]
*Sparicotyle chrysophrii* (Van Beneden & Hesse, 1863) Mamaev, 1984	*Sparus aurata* (Valenciennes)	France	AF311719	[15]
*Pagellicotyle mormyri* (Lorenz, 1878) Mamaev, 1984	*Lithognathus* mormyrus (Linnaeus)	France	AF311713	[15]
*Atrispinum acarne* Maillard & Noisy, 1979	*Pagellus acarne* (Risso)	France	AF311702	[15]
*Bivagina pagrosomi* (Murray, 1931) Dillon & Hargis, 1965	–	–	AJ243678	Direct submission
*Metamicrocotyla* sp.	*Osteomugil ophuyseni* (Bleeker)	China	MH700260	Direct submission
*Polylabris sillaginae* (Woolcock, 1936)	*Sillaginodes punctatus* (Cuvier)	Australia	GU289509	[7]
*Polylabroides* sp.	*Acanthopagrus schlegelii* (Bleeker)	China	MH700258	Direct submission
*Microcotyloides incisa* (Linton, 1910) Fujii, 1944	*Rhomboplites aurorubens* (Cuvier)	USA	KU527427	[9]
*Microcotyloides incisa* (Linton, 1910) Fujii, 1944	*Lutjanus griseus* (Linnaeus)	Mexico	MG586861	Direct submission
*Caballeraxine* sp.	*Leiognathus Equulus* (Forsskål)	China	MH700261	Direct submission
*Polynemicola* sp.	*Polynemus sextarius* (Bloch & Schneider)	China	MH700265	Direct submission
*Kahawaia truttae* (Dillon et Hargis, 1965) Lebedev, 1969	*Arripis truttacea* (Forster)	Australia	GU263831	[7]
*Probursata brasiliensis* Takemoto, Amato & Luque, 1993	*Oligoplites* sp.	Brazil	AF382049	[32]
*Cemocotyle carangis* (MacCallum, 1913) Sproston, 1946	*Caranx latus* Agassiz	–	MG984598	Direct submission
*Gotocotyla bivaginalis* (Ramalingam, 1961) Rohde, 1976	*Scomberomorus commerson* (Lacepède)	Australia	AF382039	[32]
*Gotocotyla secunda* (Tripathi, 1954)	*Scomberomorus commerson* (Lacepède)	Australia	AF382040	[32]
*Neomicrocotyle pacifica* (Meserve, 1938) Yamaguti, 1968	*Caranx hippos* (Linnaeus)	Mexico	AF382043	[32]
*Bilaterocotyloides carangis* Ramalingam, 1961	–	India	KF804032	Direct submission
*Metacamopia oligoplites* Takemoto, Amato & Luque, 1996	*Oligoplites* sp.	Brazil	AF382038	[32]
*Allodiscocotyla diacanthi* Unnithan, 1962	–	India	KF804033	Direct submission
*Polystomoides asiaticus* Rohde, 1965	*Cuora amboinensis* (Daudin)	–	Z83008	[24]

## Results

### Molecular analyses

The partial 28S rDNA gene sequence of *Paracaesicola nanshaensis* n. sp. was aligned with 28 other monogenean sequences, including 19 microcotylid sequences. There was a total of 855 positions in the final dataset, including 381 conserved sites, 474 variable sites, and 338 parsimony informative sites. The Kimura-2 parameter genetic distances between our new sequences and other microcotylid sequences ranged from 13.6% to 31.3% (Table 2). The most closely related species to *P. nanshaensis* n. sp. were *Diplostamenides sciaenae* (FJ432589), *Diplostamenides* sp. (MH700263), and *Cynoscionicola branquialis* (AF382050), with estimated genetic distances (Kimura 2-parameter) of 13.6%, 13.8%, and 14.5%, respectively.

**Table 2:** Distances between microcotylid taxa (Kimura-2 parameter model), shown as percentages.

	1	2	3	4	5	6	7	8	9	10	11	12	13	14	15	16	17	18	19
2	14.5																		
3	13.8	6.7																	
4	13.6	7.4	1.1																
5	15.8	12.5	13.2	13.2															
6	16.2	12.8	13.6	13.5	0.2														
7	17.0	13.3	14.2	14.2	0.6	0.9													
8	15.5	12.7	14.5	14.1	5.1	5.4	5.8												
9	15.0	14.8	13.2	13.9	9.9	10.2	10.9	11.8											
10	16.9	13.6	14.3	14.1	5.2	5.5	6.0	5.1	12.1										
11	20.8	15.8	19.0	19.4	14.9	15.4	15.8	14.1	16.6	15.1									
12	19.2	14.3	17.5	17.9	13.8	14.2	14.5	13.4	15.7	14.7	1.0								
13	31.3	28.8	29.3	29.9	26.7	27.2	27.3	28.5	29.3	30.5	31.3	28.1							
14	21.9	15.5	18.7	18.7	9.7	10.3	11.2	11.5	16.0	13.8	20.0	20.0	50.3						
15	21.8	15.8	20.0	20.0	8.6	9.1	9.9	9.8	14.4	11.9	17.2	17.4	52.2	3.5					
16	21.4	11.3	16.0	16.0	9.6	10.2	11.1	12.5	15.1	13.1	19.2	19.2	45.8	6.9	7.3				
17	16.4	13.5	15.0	15.5	13.7	14.1	14.3	14.7	14.5	15.9	16.0	15.4	26.9	17.4	18.1	15.8			
18	17.9	16.6	16.6	16.8	12.0	12.0	13.2	12.4	13.7	15.0	17.3	16.7	29.8	18.7	17.2	18.6	13.6		
19	20.2	14.9	16.3	16.5	14.3	14.7	15.5	15.2	16.0	16.9	16.5	15.5	26.7	22.5	20.3	19.6	17.0	17.8	
20	19.5	15.1	16.0	15.8	12.2	12.5	13.3	13.1	14.8	14.7	15.6	14.9	26.8	19.4	16.7	18.1	14.9	15.0	5.3

Appellations of 20 taxa are stated below. 1 *Paracaesicola nanshaensis* (MH700264), 2 *Cynoscionicola branquialis* (AF382050), 3 *Diplostamenides* sp. (MH700263), 4 *Diplostamenides sciaenae* (FJ432589), 5 *Microcotyle sebastis* (AF382051), 6 *Microcotyle erythrini* (AM157221), 7 *Microcotyle arripis* (GU263830), 8 *Omanicotyle heterospina* (JN602095), 9 *Kahawaia truttae* (GU263831), 10 *Bivagina pagrosomi* (AJ243678), 11 *Microcotyloides incisa* (KU527427), 12 *Microcotyloides incisa* (MG586861), 13 *Polynemicola* sp. (MH700265), 14 *Sparicotyle chrysophrii* (AF311719), 15 *Pagellicotyle mormyri* (AF311713), 16 *Atrispinum acarne* (AF311702), 17 *Metamicrocotyla* sp. (MH700260), 18 *Caballeraxine* sp. (MH700261), 19 *Polylabris sillaginae* (GU289509), 20 *Polylabroides* sp. (MH700258).

**Figure 2:**
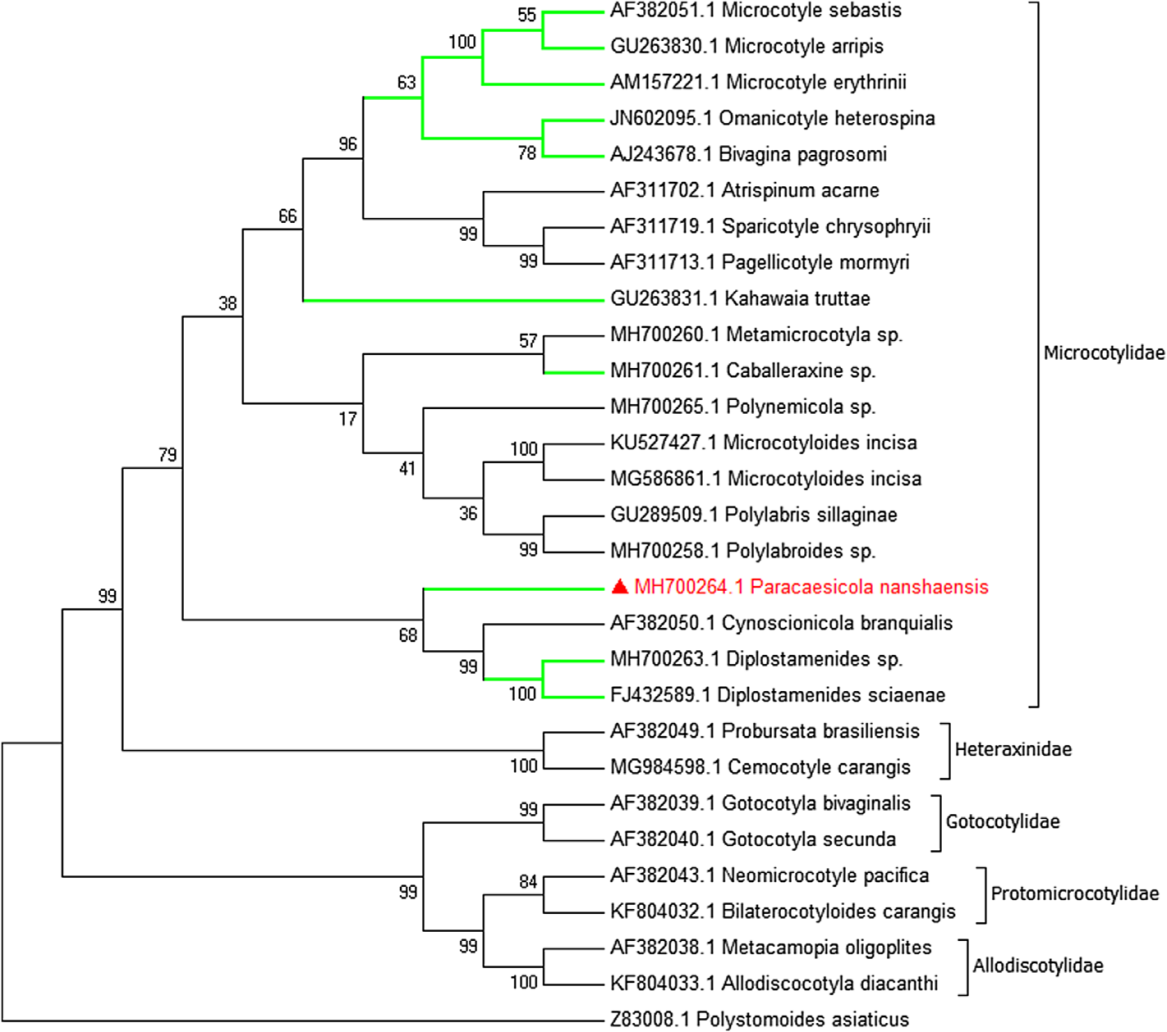
Maximum likelihood tree of the Microcotylidae based on an analysis of 28S rDNA sequences. Bootstrap percentages with 1000 replicates. The new species is in red colour and the branches of representative species in Microcotylinae are in green.

For trees, the neighbour-joining and maximum likelihood methods led to slightly different topologies. Here we present only the ML tree with Bootstrap support values at nodes (Fig. 2). The topology of the ML showed representative species belonging to different families (Microcotylidae, Heteraxinidae, Gotocotylidae, Protomicrocotylidae, Allodiscotylidae), while forming a monophyletic clade in their own family. Within the microcotylid clade, the new species *P. nanshaensis* clustered with *Cynoscionicola branquialis* AF382050 and *Diplostamenides sciaenae* FJ432589 to form a separate monophyletic subclade (68% bootstrap support in ML, 76% in NJ). However, members of Microcotylinae do not form an exclusive clade in their own subfamily. The molecular phylogenetic results supported *P. nanshaensis* n. sp. as a new taxon.

### *Paracaesicola* n. gen.

urn:lsid:zoobank.org:act:B25B37E4-A32D-4952-A5B9-F249CB1FE2CD

Class: Monogenea Carus, 1863

Family: Microcotylidae Taschenberg, 1879

Subfamily: Microcotylinae Monticelli, 1892

*Diagnosis*: Body, bottle-shaped. Haptor, short, inverted triangle. Clamps of microcotylid type, arranged in two equal rows. Anchors and haptoral lappet absent. Pair of buccal suckers, septate, and muscular. Oesophagus without diverticula. Intestine extending into haptor, but not confluent posteriorly. Testes arranged in two longitudinal rows and extending into the haptor. Vas deferens, a sinuous tube, arising medially from testes and terminating in a muscular male copulatory organ (MCO). MCO sausage shaped, entirely unarmed. Genital atrium armed with a crown of robust, equal-sized spines, clustered together and standing vertically on top of MCO. Single vagina well visible, bottle-shaped, immediately posterior to MCO; vaginal pore middorsal; vaginal atrium, distinctly bulbous, armed with a small tuft of spines at the posterior end. Eggs oval, with a long coiled polar filament posteriorly. Parasitic on gills of Perciform fishes.

Type-species: *Paracaesicola nanshaensis* n. sp.

Etymology: The name refers to the host *Paracaesio sordidus*.

### *Paracaesicola nanshaensis* n. sp.

urn:lsid:zoobank.org:act:9279425B-6E9A-4BD4-92C1-8D86144A72FE

Type-host: *Paracaesio sordida* Abe & Shinohara, 1962.

Type-locality: Yongshu Reef (112°58′–112°96′ N, 9°37′–9°61′ E,), South China Sea (12 April 2016).

Site on the host: Gill.

Prevalence and intensity: Three fish were examined and all were infected. We identified 21 worms across all examined fish.

Type-material: Holotype (No. NSYS2016041201) and paratypes (Nos. NSYS2016041202–12) deposited in the Fish Parasite Laboratory, College of Life Science, South China Normal University, Guangzhou, China. Two paratypes (NHMUK No. 2019.10.30.1-2) deposited in the Natural History Museum, London (NHMUK).

Etymology: The species is named after the locality, the Nansha Islands in the South China Sea.

#### Description (Figs. 3 and 4)

**Figure 3:**
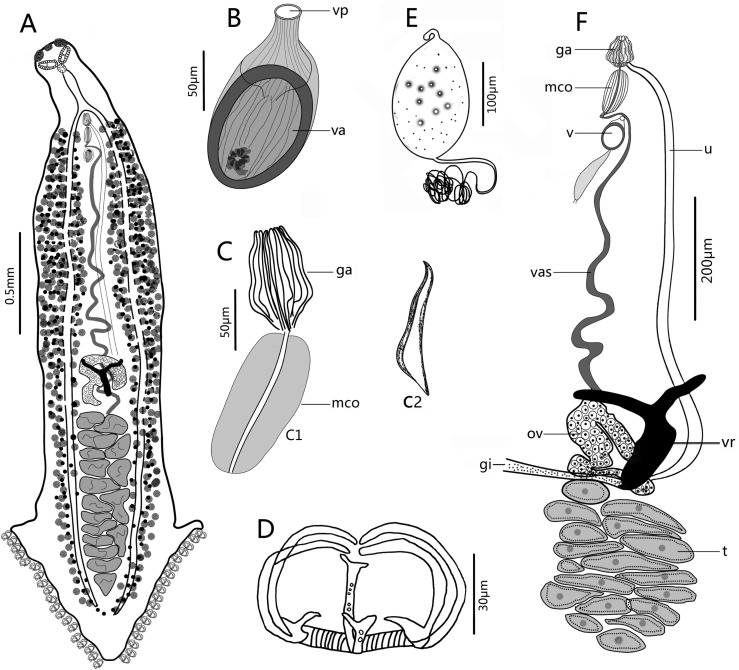
*Paracaesicola nanshaensis* n. gen., n. sp. from *Paracaesio sordida*. (A) holotype, whole body (ventral view); (B) vagina (vp, vaginal pore; va, vaginal atrium); (C) male terminal (C_1_, genital atrial spines and male copulatory organ; C_2_, a single spine in lateral view); (D) clamp; (E) egg; (F) reproductive systems (ga, genital atrial spines; mco, male copulatory organ; v, vagina; vas, vas deferens; ov, ovary; t, testes; vr, vitelline reservoir; gi, genitointestinal canal; u, uterus). B–F are paratypes.

**Figure 4:**
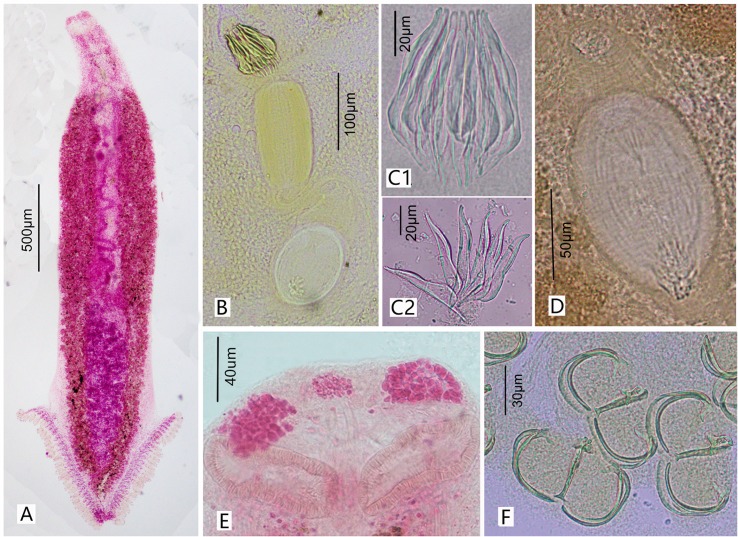
Photographs of *Paracaesicola nanshaensis* n. gen., n. sp. (A) holotype, whole worm (ventral view); (B) the terminal of the reproductive system; (C) genital atrium (C_1_ shows intact genital spines; C_2_ shows individual spines, as separated by protease K); (D) vagina; (E) head glands and buccal suckers; (F) clamps. B–F are paratypes.

(Based on 13 whole-mounted worms). Body bottle-shaped, tapering gradually from vagina towards anterior end (Figs. 3A and 4A). Body length 1986–3616 (2964, *n* = 13), body width at level of ovary 320–716 (495, *n* = 13). One median and two lateral head glands (Fig. 4E). Paired muscular buccal suckers with septa, each sucker 38–79 (50, *n* = 13) long and 78–127 (103, *n* = 13) wide. Pharynx spherical, 30–61 (47, *n* = 9) long and 31–48 (40, *n* = 9) wide, immediately posterior to buccal suckers. Oesophagus simple, without diverticula, bifurcating into two intestinal caeca at genital atrium. Intestinal caeca with lateral branches, extending into haptor, not confluent posteriorly. Haptor shaped as an inverted equilateral triangle, 613–1109 (822, *n* = 13) long and 481–1082 (755, *n* = 13) wide, bearing 55–65 (60, *n* = 13) clamps, arranged symmetrically in two bilateral rows. Clamps microcotylid type, each 30–58 (44, *n* = 22) long and 46–93 (67, *n* = 22) wide, similar in shape, with middle clamps slightly larger. Each clamp consisting of two posterolateral sclerites, two anterolateral sclerites with foot-like proximal end, and one midsclerite with dorsal and ventral Y-shaped terminations (Figs. 3D and 4F). Haptoral lappet and anchors absent. Testes 16–25 (20, *n* = 5) in number, in two roughly alternating longitudinal rows and extending into haptor (Fig. 3A), normally irregular, but presenting as a long strip in contracted samples (Fig. 3F). Testicular region 497–990 (785, *n* = 11) long, approximately one fourth of body length. Vas deferens, a sinuous tube, passing medially from testes to male copulatory organ (MCO). MCO conspicuous, well-muscled, cylindrical, unarmed, 89–150 (121, *n* = 12) long, 47–77 (58, *n* = 12) wide (Figs. 3C_1_ and 4B). Genital atrium 179–423 (327, *n* = 16) from anterior extremity of body, armed with crown of 16 robust closely packed genital spines (Fig. 4C_1_); Genital spines located immediately anterior to MCO, but not extending into MCO (Figs. 3C_1_ and 4B), with measurements as shown in Figure 1: (a) 19–31 (23, *n* = 20); (b) 49–62 (55, *n* = 20); (c) 54–68 (61, *n* = 20); (d) 72–93 (84, *n* = 20); Each spine blade shaped in lateral view and bow-like in outline, with obvious arch at the proximal third of total length, gradually narrowing anteriorly and sharply posteriorly (Figs. 3C_2_ and 4C_2_). Ovary pretesticular, resembling a question mark (Fig. 3F). Vitelline reservoir Y-shaped. Vitellarium coextensive with intestinal caeca. Single vagina well visible, immediately posterior to MCO, bottle-shaped (Figs. 3B, 4B, and 4D); vaginal pore opening middorsally; vaginal atrium oval with a hardened, muscular shell, 106–131 (116, *n* = 12) long, 67–103 (84, *n* = 12) wide, armed with a small tuft of irregular spines at the posterior end. Occasionally, a long bag-shaped muscular canal linked to posterior vaginal atrium. Uterus a slender tube, extended anteriorly into genital atrium. Genitointestinal canal visible in some specimens, enters right intestinal caecum (Fig. 3F); oviduct, oötype not observed; precise junctions between vitelline reservoir, ovary, uterus, and genitointestinal canal not elucidated. Eggs oval, 107–167 (134, *n* = 12) long and 45–103 (76, *n* = 12) wide, with two filaments; anterior one short, posterior one very long and coiled (Fig. 3E).

### Differential diagnosis

Based on the revision of Mamaev [29], we here assign the new species to the Microcotylinae by the following morphological features: a symmetrical haptor with microcotylid-type clamps, armed genital atrium, intestinal caeca unfused posteriorly, absence of terminal lappet and anchors, a pretesticular ovary in the form of a question mark, numerous testes, a single middorsal vagina. The new genus is erected herein primarily on the basis of the morphological features of terminal structures of the reproductive system: the sausage-shaped muscular MCO, the armed genital atrium, and the bottle-shaped vagina with distinctly bulbous vaginal atrium.

As suggested by Yoon et al. [39], genera within Microcotylinae can be distinguished by the number of vaginal openings, armature of the vagina, and of the male terminal. Among Microcotylinae, *Paracaesicola* n. gen. most closely resembles *Atriostella* Unnithan, 1971 as indicated by the armed genital atrium and unarmed MCO [39]. However, it can be distinguished from it by the haptor, the MCO, and genital spines [34]. The haptor of species of *Atriostella* spp. is slender tail-like, slightly less than half total body length, the genital atrium is armed with usually long spines of dissimilar lengths, and the cirrus is roughly spheroidal with lobes. Moreover, the vagina of *Atriostella* spp. is unarmed. All these features are highly dissimilar to those in the new species described.

## Discussion

Within Microcotylidae, the armatures of male and female terminalia are complex and diverse. *Paracaesicola nanshaensis* n. g. n. sp. is characterised by its sausage-shaped muscular male copulatory organ (MCO) and bottle-shaped vagina. Except for species in *Microcotyloides* Fujii, 1940 and *Polynemicola* Unnithan, 1971, the MCOs of most microcotylid species are conical in shape, bulbous, or not differentiated [9, 29]. In addition, the unique arrangement of the genital spines, MCO, and vagina in *P. nanshaensis* n. sp. (closely clustered in a line, such that the vagina is located immediately posterior to the MCO, and the MCO is also immediately posterior to the genital spines) and the disposition of genital atrium spines (tightly gathered and standing vertically on top of the MCO) are not seen in the other microcotylid species (Fig. 4B). Moreover, in *P. nanshaensis*, testes extend well into the haptor. In all other microcotylid species with a separate haptor, there is little to no overlap between the haptor and the testicular field [23, 36]. Morphological characters are, however, often affected by sampling and fixation conditions, as has been observed in studies of other species of Platyhelminthes [1, 2]. Here, the samples placed directly in alcohol exhibited a strong contraction of the body, causing the testes to transform from a normal follicular shape into a long transverse strip. Machkewskyi et al. [26] identified seven measurements in microcotylids as independent of body length: pharynx length, genital atrium length, vitello-vaginal duct length, number of testes, number of clamps, length of the clamps, and width of clamp. The size of the haptor is determined by the number and the size of clamps. In *P. nanshaensis*, the haptor bears relatively few clamps (55–65). As a consequence, the haptor of *P. nanshaensis* is short and presents as an inverted equilateral triangle. Some microcotylid species have approximately the same or fewer clamps, as compared to *P. nanshaensis* (e.g., some species in *Metamicrocotyla* Yamaguti, 1953, *Microcotyle* Van Beneden & Hesse, 1863, *Polylabris* Euzet & Cauwet, 1967 and *Solostamenides* Unnithan, 1971) [2, 12, 21, 36]. However, the number of clamps in microcotylids varies greatly, even within the same genus.

The erection of this new genus was further supported by our molecular phylogeny, which placed *P. nanshaensis* in the microcotylid clade, but with a high genetic difference from all other ingroup microcotylids. Our phylogenetic topology was congruent with previous phylogenetic studies that recovered *Omanicotyle heterospina* grouping with *Bivagina pagrosomi*, and further forming a clade with *Microcotyle* spp. [16, 32, 39]. This clade has a relatively close relationship with species in the subfamily Atriasterinae (*Sparicotyle* Mamaev, 1984, *Pagellicotyle* Mamaev, 1984 and *Atrispinum* Euzet & Maillard, 1974) [15]. However, *Paracaesicola nanshaensis* do not share a recent common ancestor with other members of Microcotylinae. Additionally, other members of the subfamily Microcotylinae do not form an exclusive clade*. Diplostamenides* Unnithan, 1971 and *Caballeraxine* Lebedev, 1972 (Microcotylinae), for example, group with *Cynoscionicola* Price, 1962 (Anchoromicrocotylinae Bravo-Hollis, 1981) and *Metamicrocotyla* (Metamicrocotylinae Yamaguti, 1963), respectively. This result suggests that the Microcotylinae maybe require division into smaller subfamilies. The phylogeny of the Microcotylidae is not well resolved, because the bootstrap values in some branches are low. Owing to the paucity of molecular sequences for species in the Microcotylidae, it is difficult to accurately determine phylogenetic relationships within its families. Therefore, sequence data for additional taxa are required.
